# Views of Swedish Commissioning Parents Relating to the Exploitation Discourse in Using Transnational Surrogacy

**DOI:** 10.1371/journal.pone.0126518

**Published:** 2015-05-08

**Authors:** Anna Arvidsson, Sara Johnsdotter, Birgitta Essén

**Affiliations:** 1 Department of Women’s and Children’s Health, International Maternal and Child Health, Uppsala University, Uppsala University Hospital, Uppsala, Sweden; 2 Department of Health and Welfare Studies, Malmö University, Malmö, Sweden; Center for Human Reproduction, UNITED STATES

## Abstract

Transnational surrogacy, when people travel abroad for reproduction with the help of a surrogate mother, is a heavily debated phenomenon. One of the most salient discourses on surrogacy is the one affirming that Westerners, in their quest for having a child, exploit poor women in countries such as India. As surrogacy within the Swedish health care system is not permitted, Swedish commissioning parents have used transnational surrogacy, and the majority has turned to India. This interview study aimed to explore how commissioning parents negotiate the present discourses on surrogacy. Findings from the study suggest that the commissioning parents’ views on using surrogacy are influenced by competing discourses on surrogacy represented by media and surrogacy agencies. The use of this reproductive method resulted, then, in some ambiguity. Although commissioning parents defy the exploitation discourse by referring to what they have learnt about the surrogate mother’s life situation and by pointing at the significant benefits for her, they still had a request for regulation of surrogacy in Sweden, to better protect all parties involved. This study, then, gives a complex view on surrogacy, where the commissioning parents simultaneously argue against the exploitation discourse but at the same time are uncertain if the surrogate mothers are well protected in the surrogacy arrangements. Their responses to the situation endorse the need for regulation both in Sweden and India.

## Introduction

Many studies have found infertility to be very stressful. Involuntarily childless people are usually completely focused on various options for acquiring a child [1, 2] and preferably one who is biologically related to them [3]. Increasing access to assisted reproductive technologies (ART) has opened new path to obtaining genetically related children. Although gestational surrogacy has been around since the 1980s, it is still controversial because it requires the physical participation of a third party who carries the child for the couple. Due to disparate country-specific legislation on surrogacy and varying access to infertility treatments, cross-border reproductive care (also known as reproductive tourism) is a growing phenomenon [4, 5].

Discrepancies in the regulation of surrogacy show its contested status as a reproductive method. In many industrialised countries surrogacy is banned (e.g. France, Germany, Italy, Spain, and some states in the U.S.), while other countries allow altruistic surrogacy or impose certain regulations on the practice (e.g. the U.K., the Netherlands, Greece, Australia, and South Africa) [6, 7]. In Sweden, surrogacy is partly unregulated but this situation is under review by the government and might change [8]. Israel has created an internal state-funded market in which only Israeli commissioning parents are permitted to have children through Israeli surrogate mothers, thus disallowing reproductive tourism [9]. California is unique as an area where surrogacy arrangements are organised through agencies with little state interference [6]. India is well known for having turned surrogacy into a multi-million dollar market serving couples from all over the world [10–14]. It has accordingly been called ‘a transnational hub for surrogacy’ ([15] p.14).

To a great extent the international debate on surrogacy revolves around the concept of exploitation. As used here, we define exploitation as ‘the taking of unfair advantage such that one individual or party gains at another’s expense’ ([15] p.25). Critics of commercial surrogacy in a global reproductive market highlight inherent power inequalities between commissioning parents and surrogate mothers [13, 16, 17]. Transnational surrogacy has therefore been described as a ‘reproductive supermarket’, in which ‘industrial wombs’ or ‘outsourced wombs’ are marketed and ‘baby-selling’ takes place (e.g. [18, 6]). Critical voices contend that the global fertility market is structured along class, ethnic, and racial lines, and that capitalist logic leads to exploitative relationships between poor women in low-income settings and wealthy commissioning parents [6, 11, 19]. Also in Sweden this view is notable in the media, especially among feminists. There is, however, no unified feminist position, either internationally or in Sweden: some feminists condemn all forms of surrogacy as a commodification of women’s reproductive capacity and as exploitative per se, while others believe women should have the democratic reproductive freedom to enter into surrogacy contracts [12, 20–25]. In India there is even a discourse describing surrogacy as an opportunity for empowerment for the surrogate mothers because of the possibility of a significant financial benefit for themselves [24, 26].

Similarly, there are divergent views on the medical consequences of surrogacy. There are some who highlight the increased risks to which surrogate mothers are exposed because of hormonal treatments, multiple gestations, and unnecessary caesarean sections [12, 27, 15]. Others report that the close medical surveillance during surrogate pregnancies makes those pregnancies less likely to bring severe complications (e.g. [28]).

The key issue being debated is whether surrogacy should be construed in terms of *opportunity* and *choice*, or *inequality* and *exploitation* [29, 23, 10, 30]. Some claim that it is a win-win situation for surrogate mothers and commissioning parents alike, in settings such as India [27]. Others argue that the relationship between commissioning parents and surrogate mothers is never purely contractual but has an inescapable ethical dimension with unequal social powers [29].

There is no legal regulation of ART in India. Only non-binding guidelines exist. According to governmental guidelines, a surrogate contract should be drawn up in which the surrogate mother relinquishes all parental rights and agrees to avoid harming herself and the baby. The commissioning parents in turn commit to paying for all costs associated with the surrogacy arrangements and the guidelines also state that the commissioning parents will have full custody of the child immediately after birth [31]. The rapid increase of surrogacy arrangements in India has, however, created a need for legislation beyond unenforceable guidelines. A government bill on ART was drafted in 2008, but has not yet been passed. It has been criticised by Indian scholars for not sufficiently protecting the surrogate mother’s health and interests [32, 33].

Transnational gestational surrogacy is under-researched [15] and there are currently only a few studies of Western commissioning parents’ experiences of using transnational surrogacy. These are mainly included in larger studies focusing on several issues of the surrogacy process [34–37], so we know little of the views and concerns of prospective parents who contract for transnational surrogacy. For the present study we interviewed Swedish commissioning parents who have decided to let women abroad carry their children through surrogacy arrangements. Sweden is conspicuous as one country where the technology enabling surrogacy is unlawful [38], but where the practice is otherwise unregulated. Hence, people who want to have a family by means of surrogacy cannot have clinic-based treatment in Sweden, but can make those arrangements on the global market. As may be expected, such a decision can involve some moral qualms. It has been argued that cross-border reproductive care may work as a ‘safety valve for couples whose moral principles are not represented by the laws regulating their own countries’ ([39] p.20). Commissioning parents who acquire their children by travelling to a country such as India might find themselves implicitly or explicitly accused of being immoral and exploitative (see e.g. Parks ‘calling commissioning couples to task,’ ([29] p.336).

The prevalent discourse on transnational surrogacy as an exploitive activity is not unknown to commissioning couples [23]. The aim of our study was to explore Swedish commissioning parents’ experiences and motivations for using transnational gestational surrogacy and how they construct and negotiate their understanding of this arrangement in the light of the exploitation discourse on surrogacy.

## Materials and Methods

### Participants

The study included fifteen Swedish couples who had used transnational surrogacy. Six couples were heterosexual; nine were same-sex male. The heterosexual couples were 36 to 44 years old (mean 41) at the time they had their children through surrogacy; the same-sex couples were 29 to 52 years old (mean 38). All the children were born between 2010 and 2014. Eleven out of fifteen couples lived in the Stockholm area. The participants were all well-educated and appeared to be financially well-off. All the participants were married and in all cases the commissioning father was also the genetic father. Only one of the women was the genetic mother. The rest utilised egg donors or, in the case of two couples, had made surrogacy arrangements in northern Europe and used the surrogate mother’s egg. Ten couples contracted for surrogates in India (among them all the heterosexual couples) and almost all used either of two agencies in Mumbai. The remaining five arranged their surrogacy in the U.S. or countries in northern Europe.

### Data collection

Semi-structured in-depth interviews were conducted with the fifteen couples between July 2012 and January 2015. The interviews took place at their homes, in seclusion in their work place or in a café, according to their choice. Informants were recruited through infertility NGOs, snowball sampling, and word of mouth. The interviews focused on motives for surrogacy and experiences of surrogacy procedures in the foreign country and Sweden, including interactions with the surrogate mother, the agency, IVF clinic, and authorities. It also focused on perceptions of the public discourses on surrogacy. Most interviews were conducted with only one member of the couple. Four couples were interviewed together and both people in one couple were interviewed separately at their request. One follow-up interview was made. All interviews were conducted by the first author (AA). The interviews, which lasted from 1 to 2.5 hours, were conducted in Swedish, recorded and transcribed verbatim. The interviews were then translated by the authors into English. With translation, nuances in the meaning of the quotations can be lost, which is a limitation to the study. However, the informants have been given the possibility to read the translated quotes, a procedure that minimises the risk of discrepancy in meanings between original and translated quotes.

### Analysis

Thematic analysis was used to elucidate patterns of meaning relevant to the study aim [40]. Within the material a certain data set was analysed, in which the commissioning parents expressed their perceptions and experiences about using surrogacy in relation to discourses in society. The transcripts were reviewed a number of times to find data related to the aim and were then coded. Codes were compared to find similarities and differences and were linked in order to create preliminary themes. Transcripts were then read once more and compared in order to establish the final themes (see Results). These were also weighed with regard to other relevant studies (see Discussion). Most of the analysis focused on the experiences of using surrogacy in India. Only a few of the commissioning parents used other countries for surrogacy and the analysis of these narratives is limited. Nevertheless, their perceptions on using surrogacy pose an important contrast to those relating to India.

### Ethical considerations

All participants were thoroughly informed about the aim of the study and gave their written consent prior to the interviews. Since surrogacy is a sensitive subject and very few people in Sweden have used it (according to the SMER report of 2013 it is estimated that 100 children had been born through surrogacy by 2012) [41] and many of them know each other through surrogacy networks, we took extra precautions to eliminate the possibility of identifying individuals through the quotations or by giving extensive background information. All informants had the opportunity to delete any quotations from their interviews before the manuscript was concluded.

The study was approved by the Regional Ethical Review Board of Uppsala, Sweden (registration number 2012/462).

## Results

Surrogacy was seen as a last resort in seeking to have a child. Many interviewees were affected by media discourses on surrogacy, especially the focus on the vulnerable situation of surrogate mothers. However, when researching surrogacy, they found other sources of information (e.g. internet forums for people using surrogacy) which brought new perspectives that counterbalanced the negative image of surrogacy provided by the media. Couples that had turned to India felt the need to take a stance, many of them critically, on the exploitation discourse. They referred to the financial compensation provided to the surrogate mother as the only opportunity for her to improve her economic situation and embark on a better life. Nevertheless, they also argued for legalising surrogacy in Sweden as that would protect the interests of all parties involved. All quotes below are from commissioning parents using India except one, where it is stated that the commissioning parent had used the U.S.

### The last resort

All interviewees described their great longing for a child. The heterosexual couples had a history of unsuccessful attempts to conceive prior to the surrogacy arrangement and reported a series of failed IVF trials. The homosexual couples had struggled with the likelihood that they might not get an opportunity to build a family. Having passed the upper age limit for adoption had closed that option for some interviewees. Others were deemed ineligible as adoptive parents, either due to their own medical history or, in the case of same-sex couples, because most countries do not accept homosexual people as adopting parents and there is almost no domestic adoption in Sweden. In general, adoption was not seen as a viable solution among heterosexual couples either, as exemplified by one woman who said ‘It’s quite difficult to adopt now. The administrator [at the adoption agency] told us, “There are no guarantees and you’ll be lucky if you get a child of any age at all’”. The interviewees underscored having exhausted all other options before deciding to try surrogacy. ‘Yes, I have done everything I can’, one woman related, ‘I reached the point where my body said “Enough, no more now”. Knowing that I’ve done all that I can, made me feel reassured about taking that decision [regarding surrogacy]’.

Surrogacy was consistently described as a last resort. The decision to have a child with the help of a third party abroad was depicted as a difficult one regardless of whether the surrogacy would take place in a Western country or in India. Extensive research on surrogacy, ranging from general discussions to specific information about clinics, had preceded the decision. In the process ethical issues had come to the fore: ‘There is scepticism in society against having children with a surrogate mother, and you do not want your children to become affected by it. We wanted to reach the conclusion that this is actually defensible and the right thing to do. So this was a big process as well’ (Man in a same-sex couple).

### Negotiating the discourse on exploitation in relation to the surrogate mother’s situation

As noted earlier, many interviewees were affected by media discourses on surrogacy, especially the focus on the vulnerable situation of surrogate mothers. However, the male partner in one heterosexual couple contrasted what he considered well-informed websites on surrogacy to the media framing it in the form of ‘horror stories’. Two men in a same-sex couple who were interviewed together described the dismay they experienced at watching a TV documentary on surrogacy that portrayed the situation at clinics in northern India as ‘shocking’ and ‘sickening’:

Then we felt like, we’re not getting into this process, so actually we put it on ice. Yes, we completely shut it out of our minds. And then a few months went by until we were babysitting another couple’s children and started thinking about it again. We began to ask around to find a clinic that was reputable and where women are taken care of in a good manner. That was our number-one priority. Sure, we wanted a child, but not at any price. It must be done in a proper way, with dignity as well.

The couple met two other couples who had been to a clinic in Mumbai, and these encounters gave them the opportunity to obtain first-hand information about the living conditions of the surrogate mothers at this clinic. It gave them a very different picture compared to what they had seen on TV.

The couples we interviewed reflected to a great extent on the negative opinion in media that often depicted surrogacy as exploitation, especially in the Indian context. India was the primary choice of many couples for financial reasons (surrogacy in the U.S. is considerably more expensive). In their reflection on the media discourses the couples that had turned to India rejected the exploitation discourse by referring to the surrogate mother’s situation:

For these [Indian] women, there are of course no alternatives. So for them it is a way to fulfil their lives. […] I believe that in Sweden you think … you would not do it here because here we have other choices—here you can actually enter the university without being wealthy, you can educate yourself, but there of course you cannot. The opportunities are so limited. […] If they then have the ability to carry a child for nine months and get a whole new life, why cannot people accept it? That is what makes me really disturbed. (Man in same-sex couple)

Some interviewees rejected the exploitation discourse by referring to the positive aspects of the surrogacy arrangement, describing the contractual relationship between surrogate mother and commissioning parents as one based on ethically acceptable terms. A man in a same-sex couple described it thus:

Oh, I really felt that it was mutual, that she did this because she wanted to give a gift, as simple as that. […] When we met the second and third times, we realised that this is really how she feels about it, and then you begin to realise that she really means this. Like, it’s nothing she does casually. She does it as a nice gesture, and at the same time we know that she will have financial security in the future, at least for a while. She had no job and wanted to be home with her kids and then perhaps get some money for it. I felt it was very fair and very correct and it never felt like taking advantage of someone. Certainly, many argue that we are rich, that we in Sweden exploit the system, but it certainly has not felt like that, never ever! (Man in a same-sex couple)

Still there were some doubts among the commissioning parents using India about the well-being of the surrogate mother. Some commissioning parents expressed worries of the surrogate mother’s physical well-being during pregnancy. Doubts were related to the extent of contact with the surrogate mother, and this contact depended on the agency, since all contact needed to be facilitated through them. However, all of the commissioning parents except one couple had met the surrogate mother and some had Skyped with her during the pregnancy. Many of the interviewees also described how they had tried to make sure that the arrangement with the surrogate mother would take place in a way that was ethically acceptable and non-exploitative. A man in a heterosexual couple put it in these words:

My biggest concern in this has been that something would happen to the surrogate mother… as it is of course not a risk-free thing. […] It is most important that all goes well for everyone involved and that there is no cheating, or that things are not done improperly. […] I asked [the surrogate mother], “Are you really sure [that you want to go ahead with this], because I think it’s okay if you change your mind; it won’t be a problem for us”, but she just said, “Don’t worry, I need you and you need me”. (Man in heterosexual couple)

Interviewees related how compensation for the surrogacy arrangement had resulted in improved circumstances for specific women: new houses, business endeavours, prospects for education, and opportunities to care for their own children at home. The arrangement was often presented as honourable labour and not exploitative, or as in the narrative below, better than labour where women work under bad conditions and do not get much pay:

I have worked with women’s rights and have done a lot of thinking before using surrogacy. I wanted it to be good. I stand by my decision to choose India before the U.S.… It would just be a burden for the surrogate mother to care for another child, so she would surely not have liked to keep it. People say it is exploitation, but it is just as much exploitation to purchase a backpack [produced by an Indian woman in a sweatshop], but people in that case are not nearly half as grateful. (Woman in heterosexual couple)

In contrast, the couples who had carried out surrogacy arrangements in Western countries did not spontaneously relate to the exploitation discourse and instead talked about how the surrogate mother did it for altruistic reasons (while also receiving money), wanting to help a childless couple. They also talked about the good and close relationship they had had and some still had with the surrogate mother. This was encouraged by the agency in the U.S. and also facilitated by a mutual language (the Indian surrogate mothers rarely spoke English). The reasoning about the surrogate mother was somewhat different for commissioning parents using India. The socioeconomic vulnerability of an Indian surrogate mother was emphasised and given as a reason to choose her before a woman in a more financially secure situation:

Instead of letting a woman in Sweden have that fee, I felt that I’d rather give that money to an Indian woman who has two children that she wants to send to college. That feels much more ethically correct. (Man in same-sex couple)

Despite the existence of this kind of narratives, many also favoured the possibility of having the arrangement made in Sweden.

### Advocating for legalisation of surrogacy in Sweden

Even though those we interviewed argued that as far as they knew their arrangements had turned out well for all involved, some participants still had concerns about the practice of transnational surrogacy and would have preferred the opportunity of going through a similar process in Sweden:

We didn’t do any follow-up to see whether the woman’s husband just took the money and left … We have no idea, so no one would be happier than us if we had been able to do it in Sweden in an orderly manner. That would, of course, have been good, if an ordinary Swedish woman had said, “Well, yes, I have had my three children”, and then we could go to the hospital and arrange everything. That would have been much better than all those agents and intermediaries, which is of course not optimal. (Man in a heterosexual couple)

Another participant, who used the U.S., stated that while the surrogacy process might work out well abroad, it might also end badly and should therefore be permitted with regulation in Sweden in order to protect all parties involved:

If it is done properly, it can turn out well. […] But it can also be done in the wrong way. As it works now, I think there is a big risk that it could turn out very wrong for all parties concerned. […] It is important for the surrogate mother that it be properly regulated. She gives away the most precious gift one can give to someone else, so she should not have to fear that she will suffer because of this, or that the child she gives birth to will end up in some situation that it was not supposed to. That’s why it is so important for Sweden to thoroughly investigate this issue and come to a conclusion on how to handle it. (Man in a same-sex couple)

These narratives indicate that although the participants rejected the exploitation discourse based on their own experience of using surrogacy, some commissioning parents still had concerns regarding the surrogate mothers’ medical and social situation, and therefore argued for legalisation of surrogacy in Sweden. A man in a homosexual couple who used India claimed that too little attention is given to the legal protection of surrogate children and the women giving birth to them.

## Discussion

Transnational commercial surrogacy brings out the issue of potential exploitation in light of global socioeconomic inequalities. From the perspective of commissioning parents the practice involves moral qualms and is perceived as a last resort. Interviewees were aware of the exploitation discourse but defied it on the grounds that the Indian surrogate mothers wanted to do this in order to improve their life situation through the financial benefit of the surrogacy arrangement. Instead of commissioning parents seeing their actions as part of an exploitative global business, they focused on the welfare of the individual woman.

The commissioning parents justified their use of surrogacy as contributing to a significant benefit for both parties in the surrogacy arrangement. This kind of narrative is also found in another study on commissioning parents’ perceptions of using transnational surrogacy in India, and is interpreted as contradicting the discourse of exploitation [36]. Similar views on surrogacy are found among agencies in India, emphasising the great financial benefit and empowerment for the surrogate mother [42, 36]. It can then be assumed that commissioning parents in our study also have been influenced by the discourse of agencies. Still, in their contact with the surrogate mother, the commissioning parents tried to gain insight into her situation. Even though they got the impression that their surrogate mother was not exploited, some still had concerns about Indian surrogate mothers’ interests, and worried that these might not always be well protected. This is in contrast to those using surrogacy in Western countries, who had closer contact with the surrogate mother without involvement from agencies, and who did not express any uncertainties about the surrogate mother’s situation.

The ambiguous feelings towards surrogacy, that many of our interviewees explained they had felt before starting the process, are echoed in other parts of the world. Markens [23], who has researched discourses on surrogacy in the U.S., remarks that, although transnational surrogacy is often condoned because of the assumed benefit for the two parties involved, it comes with the awareness that surrogacy activities in India are in need of legal reform. To decrease the risk of exploitation, the parameters of informed consent should be tightened to protect the surrogate mother’s health and social interests. With only guidelines to follow and no regulation, adherence to good ethical medical practice exclusively relies on the ART clinics’ and agencies’ own feelings of responsibility. There are reports about clinics commissioning parents call “nontransparent and ‘fishy’” [36]. This would suggest that the non-exploitative impression that the commissioning parents report in our study might not be an impression they would have got at other clinics and agencies in India.

The agencies’ role in mediating the contact between the surrogate mother and the commissioning parents, led to uncertainties whether protection of the surrogate mother was always fulfilled. This contrasts to surrogacy arrangements in Western countries, where agencies did not have a similar role. However, the surrogate mothers in India benefit financially proportionally more from the arrangement and it can make a substantial difference in their lives at least in the short term, or in the long run for their children receiving better education. This is also an argument among some commissioning parents for choosing India. Some of the participants in our study who rejected the notion of exploitation argued that there is a lack of understanding regarding local context and the motives women have for becoming surrogate mothers. Regarding Western moral views on surrogacy in India, feminist philosopher Alison Bailey states that this approach incorrectly interprets the surrogate mothers’ experiences and ‘raise the specter of discursive colonialism’ ([10] p.716). This standpoint is consistent with that of sociologist Amrita Pande [43–47] who has conducted extensive fieldwork at a surrogacy clinic in India. She has explored the surrogate mothers’ situations and experiences of being part of transnational surrogacy arrangements and argues that the complex realities of poor Indian women must be the starting point for a nuanced debate about surrogacy [44].

Many interviewees using India describe a trajectory in which they went from a negative view of transnational surrogacy that was based on alarming mass-media reports, to more complex and nuanced positions as a result of their dealings with the surrogate mother and understanding of her life situation. This view is reflected in a study by sociologist Shamila Rudrappa, in which she found that the surrogate mothers’ situation as garment workers, prior to entering the arrangements with the surrogacy clinic, were perceived as far more exploitative than being a surrogate mother, which gave more meaning to their lives [48]. The situation of the surrogate mothers can be seen as relatively less exploitative than their former labour situation, according to Rudrappa’s findings.

Regarding the medical treatment and also the actual consequences of the surrogacy arrangement, these aspects were referred to as issues that stirred worry among some of the commissioning parents. The limited contact with the surrogate mother would make it difficult to get knowledge of the surrogate mothers’ well-being and how they are taken care of. In several studies it has been found that the surrogate mothers have not been properly informed about what kind of medical treatment they would get and the risks involved [44, 49, 36], thereby raising doubts to their informed consent [49].

The discourse on exploitation and surrogacy being an ethically questionable practice, influenced the commissioning parents initially, and led to a thorough search for a clinic or a way to minimise the risk for the surrogate mother not be well cared for. In line with this, Banerjee suggests that commissioning parents being more critical of how they use transnational reproduction would have the potential to reduce the risk of exploitation of surrogate mothers [50].

The definition or impact of exploitation is difficult to judge in an Indian context, where certain exploitation also can mean the potential of empowerment. Commissioning parents’ ambiguous views on surrogacy are mirrored in the current discussion on commercial transnational surrogacy in low-income settings, which embodies contrasting perspectives. On the one hand, some argue that ‘the only acceptable solution to international surrogacy in India remains abolition’, citing these activities as inherently exploitative [51]. On the other hand, despite incontestable power imbalances between surrogate mothers and commissioning parents, surrogacy may be a way out of poverty for many Indian women. It can present an opportunity for personal empowerment and increased independence in relation to husbands and families, a fact that complicates viewing the issue solely in terms of oppression and exploitation [50]. It has been shown that there is a strong correlation between an improved socioeconomic situation and positive health outcomes for both individuals and groups [51].‬ However, a stronger emphasis on reproductive rights on behalf of the surrogate mothers in ART clinical routines would be welcome.

The commissioning parents’ expressed uncertainty about the surrogate mothers’ interests not being well protected also led to arguments for allowing surrogacy in Sweden in order to increase the safety for all involved. The desire to reproduce makes people go to great lengths to have children [52], but regulation in the home country, allowing domestic surrogacy, would be offering an alternative of using surrogacy in a context where it could be seen as more ethically acceptable. However, there are still discourses with ethical arguments against all kinds of surrogacy in whatever context it may occur [20]. This method of reproduction would then still risk being questioned in society, and ethical issues would need to be navigated even for domestic use.

The views of commissioning parents are important for a comprehensive understanding of the surrogacy context in all its complexity. A better understanding of commissioning parents’ experiences, decisions, and motives may contribute to future efforts to make improvements in this area.

## Conclusion

Our study demonstrates the complex use of transnational surrogacy with colliding discourses. Infertile couples who have engaged in this practice also problematise the widespread exploitation discourse when referring to their encounters with surrogate mothers and the insight gained into their life situations, while they simultaneously argue for a regulation in Sweden.

With the growing phenomenon of cross-border surrogacy, the legal aspects of the mode of reproduction need to be regulated on both national and international levels. The European Society of Human Reproduction and Embryology (ESHRE) and the International Federation of Gynecology and Obstetrics (FIGO) have addressed the exploitation risk in surrogacy in their guidelines for regulating surrogacy, which can be useful in regulating efforts [53, 54]. It is also important, however, to consider commissioning parents’ experiences of using transnational surrogacy and the context-based issues of this reproductive method, as shown in this study. With the Ministry of Justice’s reviewing of the situation of surrogacy, it remains to be seen if there will be an alternative for potential commissioning parents to use surrogacy within Sweden.
